# Hurricane Evacuation Laws in Eight Southern U.S. Coastal States — December 2018

**DOI:** 10.15585/mmwr.mm6936a1

**Published:** 2020-09-11

**Authors:** Judy Kruger, Michael J. Smith, Brenda Chen, Brandon Paetznick, Belen Moran Bradley, Rosa Abraha, Marinda Logan, Erich R. Chang, Gregory Sunshine, Sandra Romero-Steiner

**Affiliations:** ^1^Division of State and Local Readiness, Center for Preparedness and Response, CDC; ^2^Office of the Director, Center for Preparedness and Response, CDC; ^3^Office of the Associate Director for Communication, Center for Global Health, CDC; ^4^Office of Science, CDC; ^5^Public Health Law Program, Center for State, Tribal, Local and Territorial Support, CDC; ^6^Office of Science and Public Health Practice, Center for Preparedness and Response, CDC.

National Preparedness month is observed every September as a public service reminder of the importance of personal and community preparedness for all events; it coincides with the peak of the hurricane season in the United States. Severe storms and hurricanes can have long-lasting effects at all community levels. Persons who are prepared and well-informed are often better able to protect themselves and others (*1*). Major hurricanes can devastate low-lying coastal areas and cause injury and loss of life from storm surge, flooding, and high winds (*2*). State and local government entities play a significant role in preparing communities for hurricanes and by evacuating coastal communities before landfall to reduce loss of life from flooding, wind, and power outages (*3*). Laws can further improve planning and outreach for catastrophic events by ensuring explicit statutory authority over evacuations of communities at risk (*4*). State evacuation laws vary widely and might not adequately address information and communication flows to reach populations living in disaster-prone areas who are at risk. To understand the range of evacuation laws in coastal communities that historically have been affected by hurricanes, a systematic policy scan of the existing laws supporting hurricane evacuation in eight southern coastal states (Alabama, Florida, Georgia, Louisiana, Mississippi, North Carolina, South Carolina, and Texas) was conducted. After conducting a thematic analysis, this report found that all eight states have laws to execute evacuation orders, traffic control (egress/ingress), and evacuation to shelters. However, only four of the states have laws related to community outreach, delivery of public education programs, and public notice requirements. The findings in this report suggest a need for authorities in hurricane-prone states to review how to execute evacuation policies, particularly with respect to community outreach and communication to populations at risk. Implementation of state evacuation laws and policies that support hurricane evacuation management can help affected persons avoid harm and enhance community resiliency (*5*). Newly emerging and re-emerging infectious diseases, such as SARS-CoV-2, the virus that causes coronavirus disease 2019 (COVID-19), have and will continue to additionally challenge hurricane evacuations.

Consistent with the principles of legal epidemiology,* evacuation laws enacted as of December 31, 2018, in southern coastal states were collected and systematically examined. State laws related to large-scale evacuation were identified using the search string SD((evacuat! Egress ((leave vacat!) /s area))) in Thomson Reuters Westlaw (Eagan, Minnesota), a subscription-based legal research service. Statutes and regulations were analyzed using an abstraction instrument with guidance from legal professionals. Each law was reviewed by two independent reviewers. Discrepancies were resolved in consultation with CDC’s Public Health Law Program.

The search identified 2,150 laws; 91 of those laws (including 72 statutes and 19 regulations) specifically addressed evacuation procedures. Domain and thematic analyses of existing laws were conducted. Seven relevant domains were identified (evacuation decision-making, communications, populations at risk, responder protection, plan agreements, transportation, and shelter) that encompassed 24 related themes informed by a literature review. Abstracted laws were collapsed into 17 relevant themes for analysis and comparison between states (Table).

**TABLE:** Coverage of state evacuation laws or policies — eight southern U.S. coastal states, December 31, 2018

Domain	Themes*	Alabama	Florida	Georgia	Louisiana	Mississippi	North Carolina	South Carolina	Texas
Evacuation decision-making	Law requires an emergency operation and evacuation plan	Y	Y	Y	Y	Y	Y	Y	Y
	Law specifies who may order an evacuation	Y	Y	Y	Y	Y	Y	Y	Y
	Law specifies a trigger for ordering an evacuation	Y	Y	Y	Y	Y	Y	Y	Y
	Law requires or recommends a plan to have provisions for mandatory or voluntary evacuation	Y	Y	Y	Y	Y	Y	Y	N
Communications to alert the public and outreach education	Law requires or recommends jurisdiction to provide notice to the public	N	Y	Y	Y	Y	N	Y	N
	Law requires or recommends jurisdiction to provide educational programs related to compliance with evacuation	Y	Y	Y	N	Y	N	N	Y
Populations at risk (e.g., inform limited English or diverse populations)	Law requires or recommends informing diverse racial/ethnic and limited English-speaking populations of evacuation plans	N	Y	N	N	N	N	N	Y
	Law requires or recommends informing diverse racial/ethnic and limited English-speaking populations of an order to evacuate	N	N	N	N	N	N	N	N
	Law requires or recommends informing persons with access and functional needs of evacuation plans	N	Y	N	Y	N	N	N	Y
	Law requires or recommends informing persons with disability or access and functional needs of an order to evacuate	N	Y	N	Y	N	N	N	N
	Law requires or recommends creation of a persons with disability or access and functional needs registry for evacuation and sheltering	N	Y	N	N	N	N	N	N
Responder protection	Law includes language related to the protection of first responders who carry out evacuation orders	Y	Y	Y	Y	Y	Y	Y	Y
Plan agreements	Law requires or recommends use of memoranda of understanding or supplemental agreements for evacuation planning	Y	Y	Y	Y	Y	Y	Y	Y
	Law requires or recommends use of memoranda of understanding or supplemental agreements for carrying out evacuation	Y	Y	Y	Y	Y	Y	Y	Y
Transportation	Law requires or recommends traffic control or egress/ingress to support civil evacuation movement as a public safety measure on highways or streets	Y	Y	Y	Y	Y	Y	Y	Y
Evacuation to shelters	Law requires or recommends jurisdictional support for evacuation shelter efforts	Y	Y	Y	Y	Y	Y	Y	Y
	Law requires or recommends jurisdictional support for shelter-in-place	N	Y	N	Y	N	N	N	N

**Abbreviations:** N = no; Y = yes.

*As reflected in established laws or policies. Additional themes not shown in the table include laws that specify details of who may order an evacuation; the response trigger; whether the evacuation was mandatory, voluntary, or partial; delivery types of public notice warnings and alerts; delivery of information to inform disproportionately affected populations; the disproportionately affected populations to inform; and evacuation and protection of first responders.

All eight states have laws in place regarding evacuation decision-making, responder protection, and agreements that include memoranda of understanding and plans for transportation and evacuation to shelters. Gaps were identified in three domains: 1) communications to alert the public and public outreach, 2) populations at risk (e.g., inform limited-English language or diverse populations), and 3) evacuation to shelters. Under the populations at risk domain, none of the states required alternative language use to inform those with limited English proficiency during hurricane evacuation orders. Only one state (Florida) authorized creation of a registry for persons with access and functional needs for the purposes of evacuation and sheltering. Laws in only three of the examined states (Florida, Louisiana, and Texas) included requirements to inform persons with disabilities or access and functional needs in general emergency evacuation plans.

Laws in all eight southern coastal states granted government officials authority to order large-scale evacuation in the event of a natural disaster (Figure). States differ on who has the authority to issue the evacuations orders. In Texas, for example, local jurisdictions are responsible for issuing evacuation orders, but only the governor has this authority in Florida, Georgia, and South Carolina.

**FIGURE:**
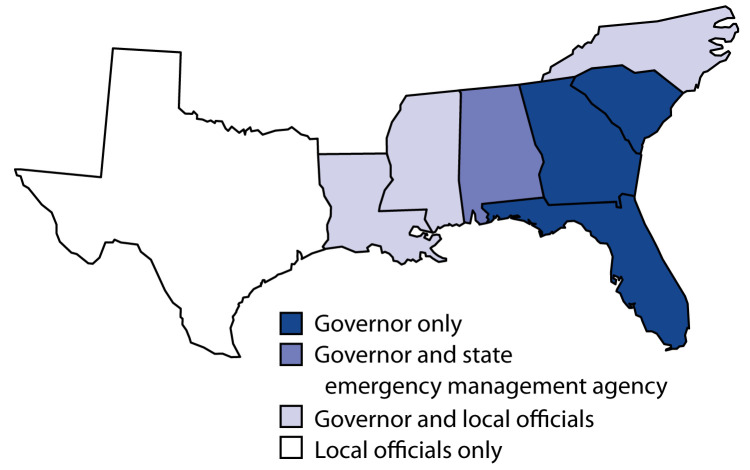
Government agencies granted legal authority to order large-scale evacuation during natural disasters — eight southern U.S. coastal states, December 31, 2018

## Discussion

Hurricane evacuations in coastal areas can prevent illness, injury, disability, and premature death by directing the movement of persons at risk out of harm’s way. Evacuation decision-making has evolved in the United States to address changing political and social environments to protect communities from anticipated hazards (*3*). Laws can further enable governments to improve planning for outreach and response to catastrophic events by specifying when and where to call for evacuations, and how to execute evacuations. Planning for hurricanes can be enhanced by providing statutory citations to communities likely to be disproportionately affected by the event when issuing evacuation orders (*4*). State officials receive hurricane awareness notifications approximately 120 hours before onset of a potential disaster event. Large-scale evacuations for natural disasters usually involve modification of major transportation routes (e.g., roadways and highways to allow rapid and orderly egress) (*6*). Evacuation plans typically commence within 72 hours before landfall; it is crucial that persons are alerted so they can safely evacuate (*6*).

Hurricane Katrina, a large Category 5 hurricane in August 2005, was one of the costliest disasters affecting the Gulf Coast, resulting in 986 deaths from drowning, injury, and trauma, and deaths among persons with chronic health conditions (*7*). Hurricane Harvey, a 2017 Category 4 hurricane, resulted in 171 deaths, including some from electrocution, car accidents, and lack of medical services (*7*). Protective actions, such as evacuation, can help keep persons alive, safe, and healthy. Lessons learned from evacuation events with poor outcomes can be used to amend and improve evacuation plans and implement relevant laws (*5*).

State laws are established to minimize the number of persons harmed during disasters and to protect those at high risk for injury and death (*8*). State governments can enact evacuation laws to protect their citizens, but successful implementation of these policies requires that state leadership effectively communicate evacuation orders and procedures to all persons in an affected area so that those persons can take action.^†^ The Association of State and Territorial Health Officials urges state government agencies to analyze their preparedness strategies on an ongoing basis (*8*). Risk perceptions, community resources, and physical ability all influence evacuation decision-making; and persons most at risk might be reluctant to evacuate (*9*). 

This report identified policy gaps, specifically in communications to alert the public and outreach education, outreach to those populations at risk because of limited English proficiency or functional and access needs, and evacuation to shelters and shelter-in-place policies. Implementation of strategies to mitigate the effect of hurricanes in coastal states through appropriate protective actions that include addressing informational needs of the whole community can minimize harm (*1*). Policies that include guidance on communication strategies for providing information to the whole community could address the identified gaps.^§^

The findings in this report are subject to at least three limitations. First, the findings do not include local ordinances or facility-specific evacuation laws, which might provide additional insight into particular evacuation powers. Second, federal mandates, which might affect legal requirements applicable to state evacuation planning, were not considered. Finally, laws by themselves are not sufficient to achieve community preparedness. Additional research is needed to understand the impact of evacuations in response to natural disasters.

States can be better prepared by providing more information about transportation routes, shelter locations, and planning for shelter supplies (e.g., adequate medications and food for persons with special needs, durable medical equipment, and generators) to address unexpected situations such as power outages lasting longer than 2 weeks (*9*). Policymakers preparing for hurricane season might develop stay-at-home policies as well as evacuation orders (*8*). Delays in recovery efforts can occur if coastal communities do not alert all persons (e.g., those aged ≥65 years, those with access and functional needs or other disabilities, and tourists). State officials should consider providing consistent messages for all media (print, radio, social media, and television) and transmitting public health messages about impending hurricanes in multiple languages and through multiple communication channels (*9*).

State officials need to analyze their strategies for evacuation policies so that they address the safety and well-being of the whole community. The most difficult part of response and recovery planning is to consider the ever-changing events that might occur during hurricane season and envision scenarios to keep the population safe. In 2020, for example, hurricane season is occurring during the COVID-19 pandemic, which might complicate evacuations and recovery. Expanded communication efforts are needed for outreach to populations at risk, residents of senior centers, and persons with disabilities (*10*). Communication plans need to support evacuation orders to address the spread of COVID-19 in shelters. Governments have issued many types of declarations for hurricane evacuations and are adapting evacuation policies to address the needs of populations at risk, who are disproportionally affected by hurricanes and infectious diseases. Clear and consistent messaging on who should evacuate and how to practice social distancing at shelters while under evacuation orders might prevent potential confusion and conflict with COVID-related stay-at-home orders (*10*). In an era of frequent major storms and emerging threats, laws providing the necessary authority to order an evacuation in coastal states can also serve to promote equitable planning, outreach, education, and dissemination of evacuation orders.

**Summary**

**What is already known about this topic?**

Hurricane evacuations can prevent illness, injury, disability, and death. Policies are established to minimize the number of persons harmed and to protect those at high risk.

**What is added by this report?**

Analysis of evacuation policies in eight southern U.S. coastal states found that all have laws to execute evacuation orders. However, only four have laws that require informing racially and ethnically diverse populations and persons with disabilities and functional needs of emergency evacuation plans.

**What are the implications for public health practice?**

Evacuation laws that include communicating evacuation procedure policies for the whole community, including populations with limited English language proficiency, might help protect communities from unnecessary hurricane-related morbidity and mortality.

## Footnotes

* https://www.cdc.gov/phlp/publications/topic/resources/legalepimodel/index.html.

† Additional information on personal preparedness is available at https://www.cdc.gov/prepyourhealth, https://www.cdc.gov/cpr/readiness/hurricane_messages.htm and #PrepYourHealth on Twitter.

§ Guidance and resources for dealing with natural disasters and severe weather is available from CDC, in multiple languages and for specific groups, at https://www.cdc.gov/disasters/index.html.

## References

[1] Federal Emergency Management Agency. Neighbors. Washington, DC: US Department of Homeland Security, Federal Emergency Management Agency; 2019. https://www.ready.gov/neighbors

[2] National Oceanic and Atmospheric Administration. Hurricanes. Silver Spring, MD: National Oceanic and Atmospheric Administration; 2020. https://www.noaa.gov/education/resource-collections/weather-atmosphere/hurricanes

[3] Rubin CB. Emergency management: the American experience. 3rd ed. New York, NY: Routledge; 2020.

[4] McGinty MD, Burke TA, Resnick BA, Smith KC, Barnett DJ, Rutkow L. Legal preparedness authority for Hurricane Sandy: authority to order hospital evacuation or sheltering-in-place in the mid-Atlantic region. Health Secur 2016;14:78–85. 10.1089/hs.2015.0068 27081887

[5] Baker K. Reflection on lessons learned: an analysis of the adverse outcomes observed during the Hurricane Rita evacuation. Disaster Med Public Health Prep 2018;12:115–20. 10.1017/dmp.2017.27 28748777

[6] US Department of Transportation; Federal Highway Administration. Using highways during evacuation operations for events with advance notice: routes to effective evacuation planning primer series. Washington, DC: US Department of Transportation; 2007. https://ops.fhwa.dot.gov/publications/evac_primer/00_evac_primer.htm.

[7] Covington T. Natural disaster statistics. Austin, TX: The Zebra; 2020. https://www.thezebra.com/research/natural-disaster-statistics/#united-states.

[8] Association of State and Territorial Health Officials. Emergency authority and immunity toolkit. Washington, DC: Association of State and Territorial Health Officers; 2011. https://www.astho.org/programs/preparedness/public-health-emergency-law/emergency-authority-and-immunity-toolkit/emergency-authority-and-immunity-toolkit/

[9] Burger J, Gochfeld M, Lacy C. Concerns and future preparedness plans of a vulnerable population in New Jersey following Hurricane Sandy. Disasters 2019;43:658–85. 10.1111/disa.12350 30990925 PMC9647963

[10] Clark-Ginsberg A, Cecchine G, Fugate C, Planning for the upcoming hurricane season in light of COVID-19. Santa Monica, CA: RAND; 2020. https://www.rand.org/blog/2020/05/planning-for-the-upcoming-hurricane-season-in-light.html

